# A Versatile Machine Vision Algorithm for Real-Time Counting Manually Assembled Pieces

**DOI:** 10.3390/jimaging6060048

**Published:** 2020-06-13

**Authors:** Paola Pierleoni, Alberto Belli, Lorenzo Palma, Luisiana Sabbatini

**Affiliations:** Department of Information Engineering, Università Politecnica delle Marche, Via Brecce Bianche 12, 60121 Ancona (An), Italy; p.pierleoni@univpm.it (P.P.); a.belli@staff.univpm.it (A.B.); l.palma@pm.univpm.it (L.P.)

**Keywords:** Industry 4.0, machine vision, machine Learning, aggregated channel features detector, blob detection, smart workstation

## Abstract

The Industry 4.0 paradigm is based on transparency and co-operation and, hence, on monitoring and pervasive data collection. In highly standardized contexts, it is usually easy to gather data using available technologies, while, in complex environments, only very advanced and customizable technologies, such as Computer Vision, are intelligent enough to perform such monitoring tasks well. By the term “complex environment”, we especially refer to those contexts where human activity which cannot be fully standardized prevails. In this work, we present a Machine Vision algorithm which is able to effectively deal with human interactions inside a framed area. By exploiting inter-frame analysis, image pre-processing, binarization, morphological operations, and blob detection, our solution is able to count the pieces assembled by an operator using a real-time video input. The solution is compared with a more advanced Machine Learning-based custom object detector, which is taken as reference. The proposed solution demonstrates a very good performance in terms of Sensitivity, Specificity, and Accuracy when tested on a real situation in an Italian manufacturing firm. The value of our solution, compared with the reference object detector, is that it requires no training and is therefore extremely flexible, requiring only minor changes to the working parameters to translate to other objects, making it appropriate for plant-wide implementation.

## 1. Introduction

The spread of the Industry 4.0 paradigm [1] has led the manufacturing industry to continuously improve as a result of advances in technology. Companies have sought virtualization and decentralization through flexibility, transparency, and integration, which are some of the Industry 4.0 design principles [2]. In order to become flexible and integrated, firms collect data to feed digital copies of everything into a Cyber Physical System (CPS) [3,4]. Data collection is also beneficial for implementation of the Lean Manufacturing (LM) paradigm, which, together with the Industry 4.0 paradigm, has been listed by many researchers as management theories and practices with reciprocal synergies [5,6]. It is usually easy to gather data using one or more contemporary technologies in highly standardized contexts. However, some complex environments still exist, especially those in which human activity is prevailing, where only very advanced and customizable technologies are smart enough to perform appropriate monitoring tasks. Among these smart technologies, the Artificial Intelligence and Computer Vision (CV) branches have been gaining relevance in recent years, due to their contributions to Intelligent Manufacturing Systems [7]. In these scenarios, various CV techniques have been tested successfully by many academics [8,9]. In addition, some examples of Visual Computing technologies, such as CV, Augmented Reality, and Virtual Reality, targeted at empowering and supporting the operators of smart factories [10,11], have been proposed for the monitoring of movements and coherently managing co-bot actions, in order to make the training of new staff easier or to help in the assembly check of particularly small electronic components [12]. According to Coffey [13], the global market of Machine Vision (MV), which is the declension of Computer Vision into the Industrial domain, will grow from US$8.54 billion in 2017 to US$16.89 billion in 2026. The fortune of MV can be attributed to its characteristics: non-contact, reliable, safe, suitable for harsh environments, and designed for working long times, among other aspects. The economic value of MV runs parallel with the examples of its practical applications [14], such as object measurement, object location, image recognition, object existence detection, and object defect detection [15]. Among the variety of manufacturing-related topics related to MV technologies, we find flexible manufacturing [16,17,18]. Nevertheless, the vast majority of studies have focused on use-cases in which human interaction is absent and the objects that have to be monitored are either stationary or moved by a conveyor [19]. The objects are manually moved sometimes, but the MV algorithm exploits manual triggering for taking snapshots of the components to be inspected once placed by the operator in the correct position. However, none of the works mentioned above have described how to concretely manage human interactions inside of a framed area.

This research work comes from the necessity of an Italian manufacturing firm to have timely and reliable data about the manual assembly process, specifically the number of assembled pieces by each of the stations of ten assembly lines on the shop floor. With managerial involvement in Lean Manufacturing and Industry 4.0, the tendency is to use a kanban system to manage the material replenishment of assembly lines [20,21], in order to keep low inventories in the production lines—and in the factory in general—with the aim of reducing costs and investments locked into raw material and spare parts. This forces managers and logistics operators to have updated and reliable information of the production of assembly lines to align, in real-time, all the upstream and downstream processes in the pull production flow, such as material replenishment, procurement, shipping, and so on. Moreover, being a high-mix low-volume company, the assembly lines are usually flexible and re-configurable: every single line is in charge of the production of several product variants. The assembly sequence is composed of customer orders and frequently involves passing from one product type to another, which implies assembling some components instead of others. The availability of an incremental, reliable, and timely individual count of assembled pieces would help operators in avoiding errors due to miscounted assembled products. By doing this, assembly operators would be supported [22,23] in managing the code change, based on the current customer order. In contexts where products are moved by conveyor belts, counting through a MV system might be trivial [24,25]; however, in our scenario, assembly can be done only manually, thus making it tricky to count pieces passed from one operator to the next one. Completed pieces, which are ready to be picked up by the following operator, are placed on a small intermediate table.

This paper improves upon our previous work [26], in which we designed a Machine Vision system able to count pieces that an operator passes to the following one by placing them on an intermediate table. The previous solution was able to count the assembled pieces and partially manage the human interactions through color-based discrimination. However, we reached unsatisfactory performances, precisely caused by the unpredictable and non-standardized interactions of humans in the phases of placing and picking up the pieces. In this work, we propose a new solution which is able to analyze a video stream with pieces manually moved by an upstream assembly operator using a preliminary procedure which decides or not to further process a frame with a suitable counting algorithm. This preliminary inter-frame difference check procedure, called Motion Check, is developed to avoid processing critical frames which might cause counting errors. Since the beginning, we have aimed to be as flexible as possible, envisioning the scalability of the system at a plant-wide level, where the product mix produced is extremely high (i.e., thousands of different products), when compared to the mix produced in one line only (i.e., order of magnitude of ten product variations). For this reason, in the proposed algorithm, we exploited some basic techniques such as image pre-processing, binarization, morphological operations [27], and blob detection. In this work, we propose the use of the Motion Check method followed by the blob-based counting algorithm to build a simple, computationally fast, and yet very versatile solution which is able to count pieces, mostly based on color and morphology. We avoided testing very advanced MV solutions, based on Convolutional Neural Networks (CNN), for the counting algorithm, as they are too computationally intensive [28,29] and, given the mandatory necessity of our system to work in real-time, they likely require huge computational resources such as dedicated GPUs. Furthermore, the usage of a CNN is probably excessive in our context, as the environment is very restricted, and only hands and products interfere in the framed area. This is the reason why very advanced object detectors, such as Deep Learning-based methods, were deemed excessive in comparison to the task at hand. Then, we compared the proposed blob-based counting algorithm to a Machine Learning-based one, an Aggregated Channel Features (ACF) Detector [30] which was specifically trained to detect the product types produced on the assembly line where we tested both MV algorithms. The ACF custom object detector was taken as a reference, in terms of accuracy of detection and counting, but we must specify that it performed well in the restricted context of that assembly line only, while it fails if we extend its application to other assembly lines where the product shapes and dimensions differs from those of the training set used. On the other hand, the developed blob-based solution is not product type-dependent and works in a manner that allows its plant-wide application (i.e., for other assembly lines also).

In this work, we compare the performance of two alternative solutions equipped with Motion Check. This procedure was added as a preliminary step to the blob-based and ACF-detector counting algorithms; its use is essential to guarantee the real-time capability of the overall algorithm and its ability to deal with manual handling. We implemented the two alternative solutions into a prototype application which provides a visual interface. Therefore, the assembly operator can visualize their production in real-time and, being conscious of the order termination, can successfully manage the critical moment of changing the order. In addition, we developed a prototype of human-friendly tool which allows even non-expert operators to easily adjust system parameters to new assembly lines. This kind of tool is essential for ensuring the plant-wide level implementation of our solution; that is, in every station of every assembly line.

The remainder of the paper is organized as follows: the detailed description of the context of use, of the system setup, and of the Motion Check procedure together with the counting algorithms are presented in the Materials and Methods section. The test setting, performance metrics, and test outcomes are detailed in the Results section; while the comparison, reasoning, and the comprehensive picture are featured in the Discussion section. In the Conclusion section, we briefly recap the entire work, and introduce the next steps we envision for the future of the project.

## 2. Materials and Methods

### 2.1. System Setup: Hardware Architecture

**Figure 1:**
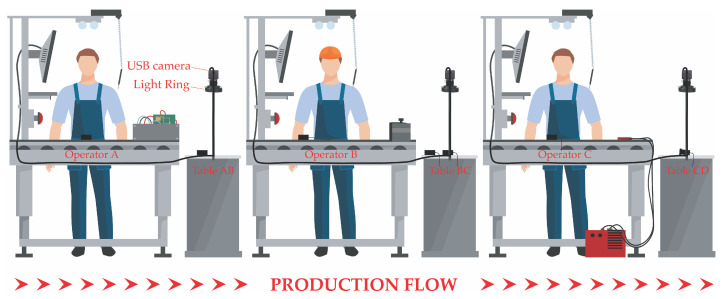
Detailed configuration of the ideal system set up in one sample assembly line.

After assessing the technological feasibility of our intention to exploit a video stream for counting pieces placed on a table, and taking into account the final aim of the company—aimed at monitoring every station of tens assembly lines—we decided to use a low-cost USB camera as the sensing device. As shown in Figure 1, every workstation of each assembly line is followed by an intermediate bench, where assembled pieces which are ready for the next step are placed. Each of these intermediate benches should be equipped with the USB camera to continuously acquire frames, which are instantly analysed by the developed algorithm in order to count the number of pieces assembled by the upstream operator.

**Figure 2:**
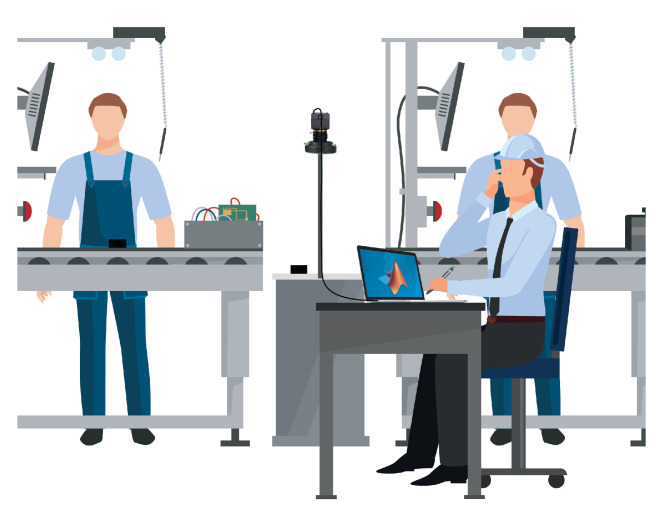
System setup used for project development.

In particular, we selected a SVPRO USB camera, with 2.8–12 mm varifocal lens, minimum illumination 0.01 lux, a Sony IMX322 sensor, 1920 × 1080 resolution, and which reaches a rate of 30 fps and uses the H.264 compression standard. For the development of this work, we focused on one camera only, acknowledging the likeness of video sequences captured by cameras framing the intermediate bench of every assembly station, as can be understood by looking at Figure 1: when acquiring in the bench AB between operators A and B, or in the bench BC, only the product shape changes, but the actions made by the upstream and downstream operators are basically the same. Going into the details of the system set up, we positioned the camera lens 80 cm away from the intermediate table plane, which is a white rectangle of 30 cm by 65 cm, along the perpendicular line passing through the table center, in order to frame a little surplus; which we can later reduce to the Region of Interest (ROI) using the computer interface. With respect to the setup used in our previous work, we added a light ring around the camera lens, in order to reduce the multiple light variations inside the assembly department due to the mixing of artificial light (which is stable and constant) with natural light from outside (which is extremely variable during the day and depends on the weather). The added light source is diffused and was produced in-house by the company. Given the prototypical nature of the study, we used our laptop, a MacBook Pro with an Intel Dual Core i7 at 2.8 GHz, 8 GB RAM, 512 MB Intel HD Graphics 3000, and 512 GB SSD storage, for carrying out all the activities. To interact with the USB camera, develop the algorithms, build the real-time processing and visualizing application, build the parameter setting application, and perform offline and real-time tests, we used the *Matlab ver. R2019a Update 7-9.6.0.1307630* software, specifically equipped with the Image Acquisition Toolbox, Image Processing Toolbox, Computer Vision Toolbox, Statistics and Machine Learning Toolbox, Matlab Compiler, and the Support Package for Generic Video Interface installed. The provisional restricted framework in which we advanced the algorithm development, data acquisitions for testing, real-time testing, and other activities described in this paper is depicted in Figure 2.

### 2.2. Context Description and Analysis

According to the system setup depicted above, if we take a typical video sequence from the camera installed over bench AB of Figure 1, which is the only one present in Figure 2, under normal working conditions of the assembly operators, we are able to discern four main phases:

1. the table is empty (*Standstill interval*);
2. the hand of operator A holds a piece, places it on the table and then goes away from the ROI of the camera (*Motion Interval*);
3. the placed piece is alone on the table (*Standstill interval*); and
4. the hand of operator B enters into the ROI and picks up the piece to take it away from the field of view (*Motion Interval*).

It may happen that a piece is placed on the table while the previous one has not been yet picked up from the following operator, which is why the solution must be smart enough to take this into account and operate efficiently during this eventuality. The Machine Vision algorithm should be able to count all the pieces placed by operator A as soon as they are put on the table, while not counting when a piece is picked up by operator B. The core of the algorithm is the object detection phase, which triggers the comparison of detected objects with already present objects and eventually decides whether to count. According to [24], object detection and consequent counting can be performed through a variety of techniques, from basic ones like filtering, morphological operations, and contrast enhancement, to more advanced solutions such as segmentation and classification models. Most of the time, the concatenation of multiple techniques proves to be beneficial. The decision of the best possible solution depends strictly on the specific characteristics of the context of use. It is, therefore, mandatory to deepen the description of the environment in which our solution is going to work, before introducing the developed algorithm. Every product type and their variations, when assembled, are partly black, while the tables are white; therefore, less-advanced image processing techniques (i.e., color-based and morphology-based ones) can work fine for solving our problem. Specifically, pieces assembled in our restricted context of project development are black-painted steel lighting devices, which are comparable to cylinders with a height of 10 cm. Several product variants are assembled in the test bed, but the cable length is the main difference among them. The validity of the presented algorithm is envisioned in the entire assembly department as well, after minor parameter fine-tuning which we make easy by using the Parameter Setting Tool application (Section 2.4.2). Indeed, different products mainly differ in dimension, but not in overall shape, which is characterized by a relevant light body with a circular, square, or rectangular section, a coloured light frame, and the cable. Broadly speaking, the algorithm can work in any context where dark-coloured objects have to be counted when placed on a white table framed by a camera. We expected more advanced techniques, such as custom-trained object detectors, to be able to learn how to detect our objects, but we also envisioned their trickier escalation into new assembly lines, due to the time required for the creation of a training data set and for the training itself of the detector. However, in a restricted context of use, a properly trained detector can be taken as reference for assessing object detection accuracy. Regardless of the kind of technique we choose to detect objects, the motion phases are the main source of error for counting algorithms. In fact, the way that people naturally and instinctively hold and move a piece during placing or picking up is extremely varied and unpredictable if not standardized or supervised. Accordingly, we tried to find an inter-frame analysis mechanism which could help us to differentiate the motion phases from the stand-still phases, in order to perform reasoning targeted at counting only during stand-still phases and not during the unforeseeable motion phases.

### 2.3. Improved Algorithms

#### 2.3.1. Motion Check

**Figure 3:**
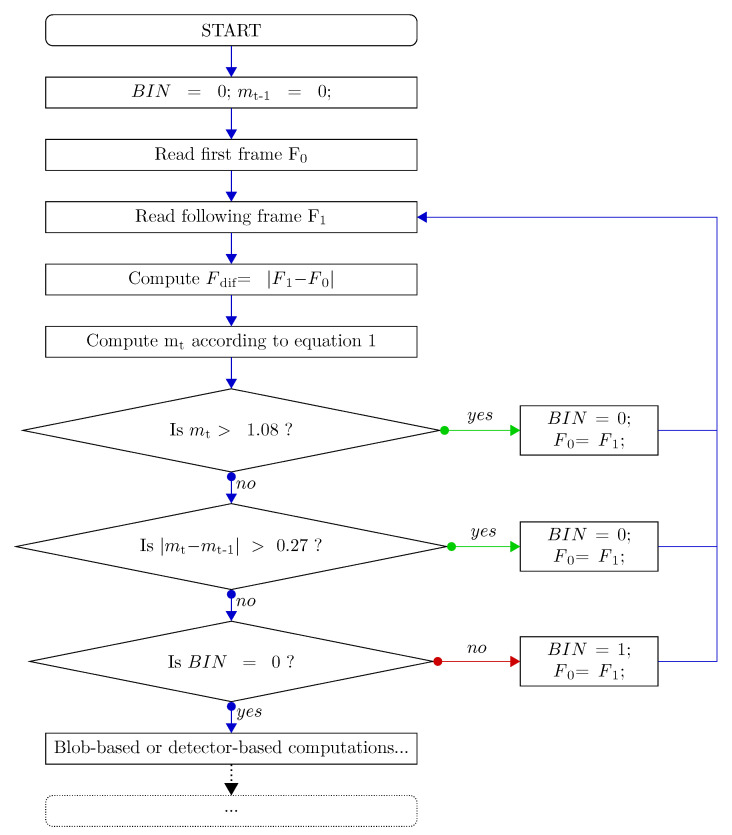
Detailed flowchart of the Motion Check algorithm.

To begin with, we present the initial Motion Check procedure, which was added as preliminary step to both blob-based and detector-based processing algorithms (which are described comprehensively later in Section 2.3.2 and Section 2.3.3, respectively). The Motion Check procedure makes the algorithms sensitive only to the eventuality that both operators simultaneously interact with the bench. In detail, when one operator places a piece while the other is picking up another piece, the algorithm (incorrectly) does not count, due to the numeric balance obtained by adding and subtracting one piece at once during the same motion interval. Nevertheless, this is a human behaviour that can be standardized after brief training of the staff, but strongly improves the counting performances of the algorithms, thus being a fair price to pay (according to the company). Every other kind of unusual product handling behaviour during placing and picking (e.g., partly covering a piece with the hand or passing over a placed piece, thus obstructing the product from the camera view for a moment), does not affect the performance of the counting algorithms provided with Motion Check. Thanks to this exploratory procedure, we avoid the need for analysing every video frame, which is computationally intensive and error-prone, due to the unpredictable placing and picking up behaviours of operators. The idea of Motion Check comes from a variety of examples found in the literature which have used background subtraction to identify moving objects [31,32]. Without going into the details of these established methodologies, we understand that comparing one frame (F_1_) with the previous one (F_0_) (specifically, by subtracting them pixel-by-pixel as in Equation (1) ), we obtain the difference image, which is different from zero in every pixel engaged in capturing something that is moving.

(1) $$p_{diff}\left(i,j,k\right)=\left|p_{F_{0}}\left(i,j,k\right)-p_{F_{1}}\left(i,j,k\right)\right|,\;\;\forall i=1,2,3;j=1,\ldots ,L;k=1,\ldots ,W.,$$

where *p_F_0__*(*i*,*j*,*k*) is the value of the previous frame pixel with co-ordinates *j*,*k* in the $i^{\mathrm{th}}$ color channel and *p_F_1__*(*i*,*j*,*k*) is the value of the current frame pixel with co-ordinates *j*,*k* in the $i^{\mathrm{th}}$ color channel. Thanks to the stable lighting provided by the light ring, during the stand-still phases where either the table is empty or one or more products are placed on it, the subtraction outcome is an image with every pixel very close to zero. In other cases, where operators are interacting with the framed area—that is, placing or picking up—there are pixels in the difference image that are much higher than zero. What we compute with the previous equation is a 3×L×W matrix, which is not immediately analysable. Hence, in order to have a more direct and synthetic measure of the magnitude of inter-frame difference, we compute the mean of all pixel values of p_diff_ in all color channels, as summarized in the following equation:

(2) $$m_{t}=\frac{1}{3*L*W}*\sum_{i=1}^{3}\sum_{j=1}^{W}\sum_{k=1}^{L}p_{diff}\left(i,j,k\right),$$

where there are three RGB colour channels in the acquired frames, L stands for frame length in pixels, *W* stands for the frame width in pixels, and *p_diff_*(*i*,*j*,*k*) is the punctual value (between 0 and 255) of the pixel in the $i^{\mathrm{th}}$ colour channel with co-ordinates *j*,*k* in the difference image computed according to Equation (1). Without the need for capturing and fixing a background, which allows the detection of generic foreground objects (either still or moving), we are able to detect the frames where there is something moving with respect to the previous one, thanks to reasoning on the value of m in the current frame. Being based on the variable previous frame, this is an adaptive movement detection procedure. A detailed flowchart of the steps involved in the Motion Check procedure is depicted in Figure 3.

Specifically, thanks to *m_t_*, *mt*−1, and BIN, which is a binary variable useful for managing the further processing as will be deepened in the Motion Check Parameters and Thresholds Paragraph, the Motion Check enables further processing aimed at counting, which is computationally intense only in two cases:

- In the first frame of a stand-still interval, just after the end of a motion phase, where BIN is equal to 0 and *m_t_* is less than 1.08; and
- In the image just after two frames within a stand-still phase which have a difference between their m values, (respectively, *m_t_* and *mt*−1) higher than 0.27.

Analysing the first case, should m_t_ go beyond the threshold, it means something is moving. As soon as *m_t_* returns within the threshold, we are again in a stand-still phase, such that we can process the frame to understand whether a piece has been added. The second case takes into consideration the eventuality that an operator moves very slowly, thus causing a small increment in the *m_t_* value which does not exceed the threshold at 1.08. In this eventuality, the BIN variable is set to 0, allowing the further processing of the following frame captured if *m_t_* will be again under 1.08. Otherwise, Motion Check never permits further image processing: if neither of the two conditions are met, it is implied that nothing has moved and no piece was placed or picked up. Alternatively, it indicates that we are inside a motion interval and there is human interaction in the framed area that can cause incorrect counting in the case of further processing of that frame; thus, further processing is prevented by the Motion Check.

##### Motion Check Parameters and Thresholds

**Figure 4:**
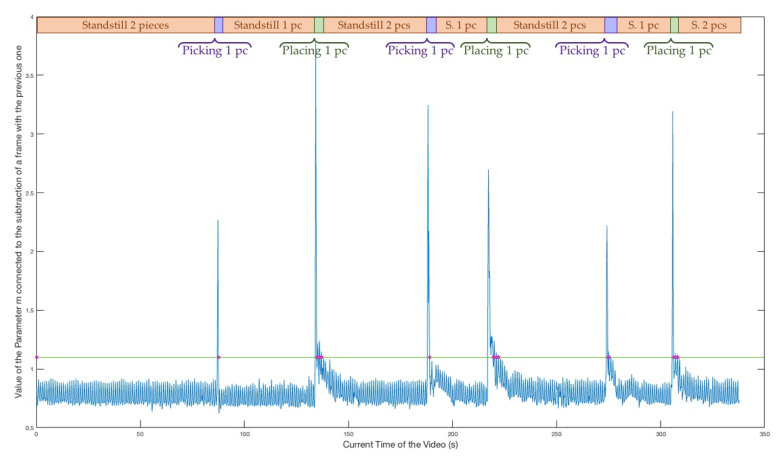
The parameter m at each instant of a sample video.

As can be derived from the flowchart, there are three main quantities relevant to the algorithm: The BIN variable is a binary one, which is useful in allowing the further processing of the first frame of a stand-still phase and to avoid processing the following frames of the same stand-still phase. This is strictly connected to the fact that, if we are within a stand-still interval of the video, nothing has moved and so no piece placing or picking up could have happened. Further processing these frames is a waste of computing power; therefore, it only occurs if BIN is equal to zero, which holds true only for the first frame of a stand-still phase (case A) or for the frame just after a slow movement (case B). The parameter *m_t_* is computed for the current frame, while $m_{t-1}$ is the value regarding the previous frame. This past value is necessary for the successful identification of very slow movements, which do not cause an increment of *m_t_* over the 1.08 threshold. We fixed the threshold for discerning motion phases from stand-still phases at 1.08 and the threshold for assessing slow movements through the difference between mt and $m_{t-1}$ at 0.27. These two values were statistically computed. After the selection of video intervals containing stand-still phases only, we computed *m*${}_{t}$ at each instant of these intervals. Computing the mean and the standard deviation of these values, we obtained, respectively, 0.67 and 0.13. Then, we fixed the mean plus three standard deviations as the motion threshold, and two times the standard deviation as the threshold for the gap between *m_t_* and *mt*−1. We present, in Figure 4, the typical behaviour of the *m_t_* parameter (computed according to the Equation (2)) in every moment of a sample video where three pieces were placed and three pieces were picked up.

The current video time is in the horizontal axis, while we have the value of the *m_t_* parameter at each instant in the vertical axis. The peaks and the values immediately after which differ from the stable trend of other intervals correspond to either the placing or picking up phases, while the stationary intervals are connected to the stand-still phases of the video. The description of the phase is shown in the timeline in the upper part of the picture. We outlined the 1.08 threshold by designing a straight horizontal green line and we point out, with magenta dots on this line, the video times for which the Motion Check decided to go ahead with further processing, as caused by one of the two conditions listed earlier.

**Figure 5:**
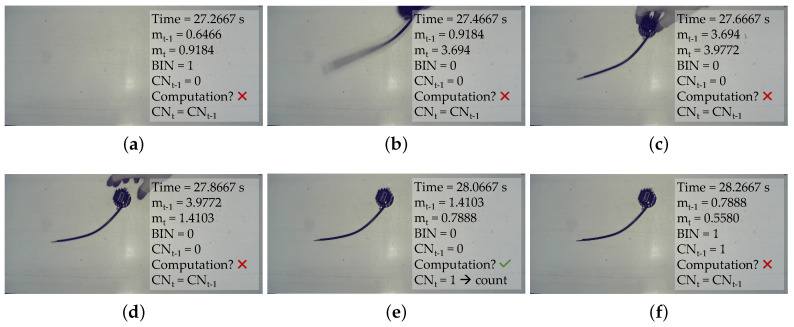
The sequence of placing one piece: (**a**) the last stand-still frame; (**b**) the first motion phase frame; (**c**) another motion phase frame; (**d**) another motion phase frame; (**e**) the first frame of a stand-still phase that is further processed; and (**f**) another stand-still phase frame, which is not further processed.

To better understand how the values of *m_t_* and *m_t−1_* affect the processing of the video frame, we collected six video frames, each 0.2 s after the previous one, that capture from the beginning of the placing of a piece through to terminating and counting. Figure 5 sums up, for every frame, the values of the relevant variables that are used by Motion Check to decide to go ahead or not with further computation, and the final decision taken by the counting algorithm which processes the frames selected by Motion Check.

In details, the first video frame shown in panel (a) is the last of the Stand-still phase, which is associated to a value of *m_t_* within the stand-still boundary. The following frame has a *m_t_* value exceeding the threshold, thus suggesting the beginning of a motion phase which lasts until the fifth frame in panel (e) that, being the first stand-still frame after a motion interval, is further analysed and returns the presence of one piece. Given that there was no previously present piece, the algorithm decides to count one. The following frame is inside the stand-still interval and the gap between the current m value and previous m value is lower than the inter-m gap boundary, and no further processing is performed on this frame. The Motion Check procedure is important, both for reducing the counting errors and improving the real-time capability of our solution, as we will elaborate in Section 3. Yet, considering that it is unable to count by itself, it is an enabler of further processing in the frames that are the most meaningful. To actually count, we need other processing algorithms which are able to detect objects and quantify how many there are in one frame. We decided to evaluate two different approaches, the first one being very simple and versatile, which is based on basic image processing techniques to identify dark objects in the framed area; while the second one is a more sophisticated machine learning-based specific object detector.

#### 2.3.2. Blob-Based Counting Algorithm

**Figure 6:**
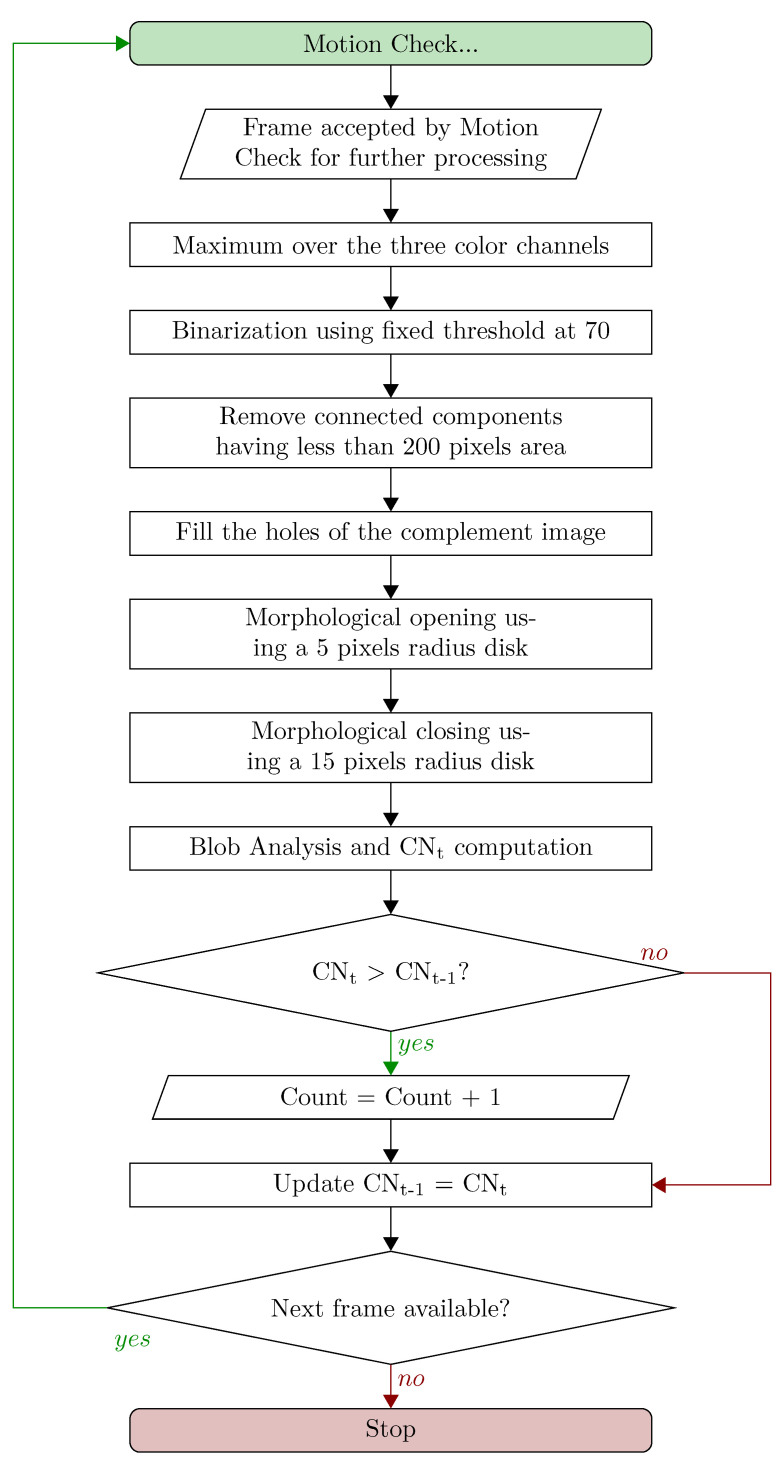
Detailed flowchart of the Blob-based algorithm.

With respect to the blob-based solution proposed in our previous work [26], we slightly changed the processing steps. Specifically, we added, in the flowchart, the removal of connected components. Moreover, we now perform image processing only when the Motion Check decides to go ahead, differing from before, where we performed the blob analysis steps for every new frame, whether it was stand-still or motion. Particularly, in the latter case, the probability to make mistakes due to the strange behaviours of operators was non-negligible and led to several hardly addressable counting errors. Motion Check overcomes these errors, as we will see in the Results section. In either case, the blob-based image processing algorithm aims to find how many dark objects there are in a given frame. A detailed flowchart of the steps required for understanding how many pieces there are in one RGB frame, according to this algorithm, is presented in Figure 6, where the first block simplifies the entire Motion Check procedure discussed extensively in Section 2.3.1.

**Figure 7:**
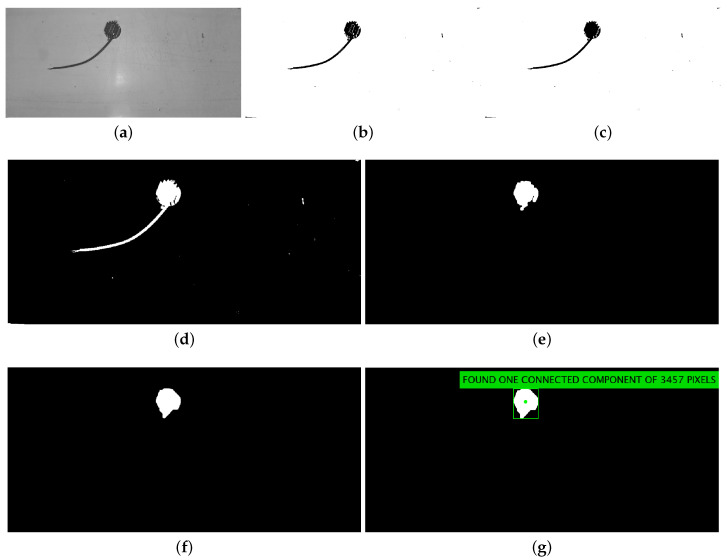
Outcome of the blob-based processing algorithm for the sample frame (**e**) of Figure 5: (**a**) maximum over the colour channels; (**b**) binarization; (**c**) small blobs removed; (**d**) filled complement image; (**e**) morphological opening; (**f**) morphological closing; and (**g**) visualization of the blob area.

The removal of connected components having an area of less than 200 pixels was added, as compared with the previous version of the counting algorithm, to better define the core of the object after binarization. In Figure 7, we show the outcome of the algorithm processing steps performed on frame (e) of Figure 5, which is the one for which the Motion Check decided to go ahead with further processing. At the end of the processing steps, we obtain the number of white blobs and some statistics like their area, centroid, and bounding box, which are annotated in panel (g) of Figure 7.

The complexity of this algorithm is connected to the definition of five key elements, such as the threshold for binarization, the radii of the disks used as structuring elements for the two morphological operations, and the minimum and maximum limits for the areas to be detected, as small white blobs due to noise or dirtiness of the table should not be taken into account when counting. Moreover, thanks to the developed Setting tool described in Section 2.4.2, even a non-expert user can change the radius of structuring elements and adapt them to diverse working conditions, while the minimum and maximum areas will be automatically computed and set by the app.

#### 2.3.3. Aggregated Channel Features Detector-Based Counting Algorithm

The alternative solution that can be launched as a counting algorithm once Motion Check decides to go ahead is a Machine Learning-based custom object detector, which was specifically trained in order to be able to detect the object of interest which passes on the test-setting bench. Specifically, we used the following training options for developing our Aggregated Channel Features (ACF) Detector: 4 stages, 176 × 165 pixels object size, 2048 maximum number of weak learners, and a training data set with 660 positive examples and one negative sample factor. Detector training took more than 13 min to be performed, but its application consists basically of two steps only, as detailed in the algorithm flowchart:

(i) Apply the detector on the frame and obtain the scores and the bounding box co-ordinates;
(ii) Examine, when more than one object has been detected, if any of these separate bounding boxes overlap (in which case, they correspond to the same object);
(iii) Compare the number of objects present in the current frame (*CN_t_*), along with the recorded number of already present objects (*CNt*−1):
  - COUNT if CN_*t*_>CN*t*−1
  - DO NOT COUNT if CN_*t*_<=CN*t*−1
(iv) Update *CN_t−1_* with *CN_t_* and go back to the Motion Check procedure with the following frame.

**Figure 8:**
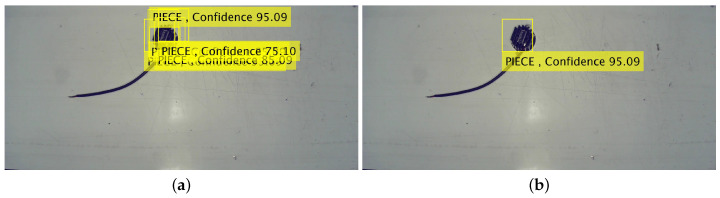
In panel (**a**) we show the multiple overlapping detection in step (i) and, in panel (**b**), the result after step (ii).

In Figure 8, we show the outputs of the algorithm processing steps (i) and (ii) performed on frame (e) of Figure 5: the detection of multiple pieces in panel (a); and the overlapping bounding box analysis, allowing us to determine all of the detected objects which are related to the same object, is shown in panel (b).

Contrary to the blob-based algorithm, the flowchart of this alternative is much shorter; however, it hides the complex and time-consuming actions which must be done before to create the detector. Specifically, we refer to the creation of an image data base for providing training data to the detector, and the time required to train the detector. In our specific case, we spent about an hour developing the image data base from videos collected over several days, while the training took 13 min. We have to specify that only a small amount of training data and basic training options were used. Furthermore, on a large scale (i.e., at a plant-wide level ), we must develop a detector for every product type and for every product perspective: in Figure 8, the piece is placed on the base but, if it were placed on its back, the detector would have failed in detecting it; or, at least, would have been inaccurate and not reliable.

### 2.4. Prototypical Implementation

The concatenation of Motion Check with either the blob algorithm or the detector-based algorithm serve as solution to the problem we are addressing, but one important thing to take into consideration is that every implementation of the solution should be able to process a real-time video stream. Therefore, we implemented both blob-based and detector-based solutions into a prototypical application.

#### 2.4.1. Real-Time Counting Application

**Figure 9:**
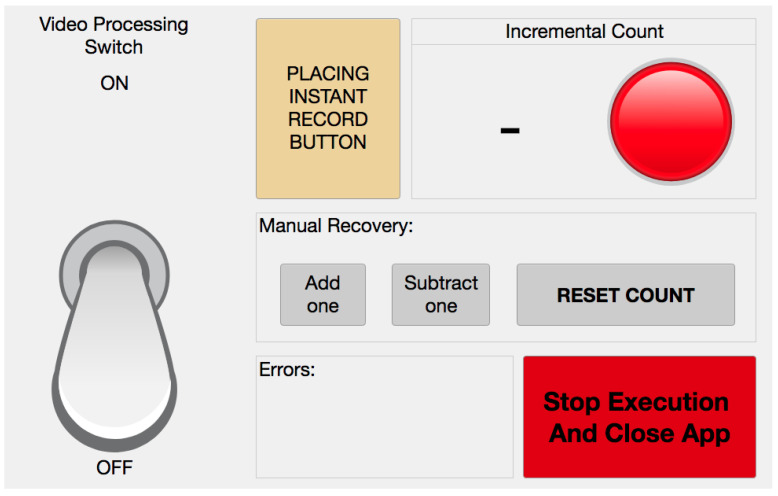
Main GUI of the prototype real-time App.

In Figure 9, we present the graphical layout of the prototype application, developed using AppDesigner in the *Matlab ver. R2019a Update 7-9.6.0.1307630* software.

While continuously processing the video stream in real time, it instantly updates the count and visualizes eventual errors, such as slow image acquisition or irregular working conditions. Starting from the top left of the picture down to the bottom right, we have the switch for managing the video processing, the “placing instant record button” to manually save the actual timestamp of piece placings, the incremental count visualized in real-time, the manual recovery buttons, the error panel, and the button to stop execution and close the app. The “placing instant record button” is essential for testing the responsiveness of our solution, as will be detailed in Section 3. The App also creates a log file that stores relevant events during working conditions. In more detail, the timestamps of every count, every frame accepted by Motion Check for further processing, every delay in image acquisition, every manual recovery, every switching of video processing, and every execution stop are registered. The log file is useful for assessing the capability of the real-time implementation of the algorithms to work for long times without losing any information due to delays in image acquisition.

#### 2.4.2. Parameter Setting Tool

**Figure 10:**
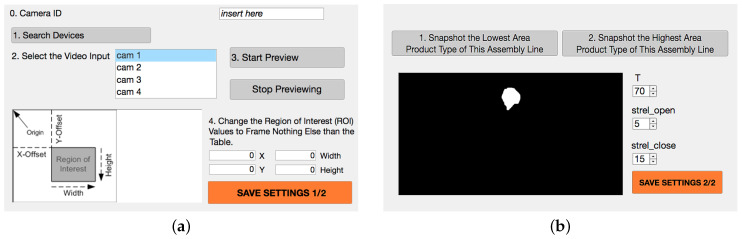
In panel (**a**), we show the first GUI of the parameter Setting Tool App and, in panel (**b**), the second GUI.

We previously introduced the importance of well-defining the value of the threshold for binarization and of the radii for morphological operations. This may be a complex task for non-experts in Computer Vision; nonetheless, to ease the adaptation of the algorithm into new assembly lines, it is essential to make this procedure fast and comprehensible. For this reason, we developed a prototype Setting Tool Application, which guides in the proper setting of the relevant working parameters. In Figure 10, its two Graphical User Interfaces (GUIs) are shown.

The first GUI allows the user to connect to a USB camera and define its ROI parameters, by visualizing the framed area with each modification of X, Y, Width, or Height. After having set parameters in the first GUI, the second one (showed in panel (b)) appears, which allows the user to see the outcome of all the blob processing steps until the morphological opening of the current situation captured by the camera. Every time that T (i.e., the binarization threshold ), the radius of the structuring element for the opening (strel open), or the radius of the structuring element for the closing (strel close) are changed by the user, the final outcome of processing, according to the defined parameters, is shown in the display. This allows the user to practically understand which modification to the process parameters improves the results of processing. The user is guided in order to be sure that the minimum area and the maximum area product are both well-defined. It is important to define these upper and lower limits for the Blob analysis, in order to avoid considering image noise or table wear as objects to be counted. Once saved, at each following utilization of the real-time processing prototype App, the set parameters are automatically used.

## 3. Results

We would like to specify that all the recorded videos and computer code are available, upon request, from the corresponding author. Our aim was to find the best solution for counting pieces assembled from an operator which places them on the intermediate table as soon as they accomplish their workload. These pieces are progressively picked up, one by one, by the following operator who carries on the assembly process. In order to effectively count the pieces manually placed by an assembly operator on a table, we developed a preliminary Motion Check procedure followed by two different counting solutions, whose performances were compared. One counting solution was designed by us, based on existing image processing techniques; while the other one is a reference method, a consolidated Machine Learning object detector. In order to objectively compare the two counting solutions, the operation managers of the company defined the following requirements that the chosen system must meet, in order to be implemented in the assembly lines of the shop floor:

(a) Count every time an assembled piece is placed on the table;
(b) Do not count whenever a piece is picked up from the table;
(c) Do not count whenever a piece is not placed on the table, in general, given that sometimes operators interfere in the framed ROI of the camera even though they are not placing nor picking up a piece;
(d) Analyse the live video stream for long times without losing any interval;
(e) Be timely in counting; and
(f) Be adaptable to all of the different assembly lines in the company’s shop floor.

**Figure 11:**
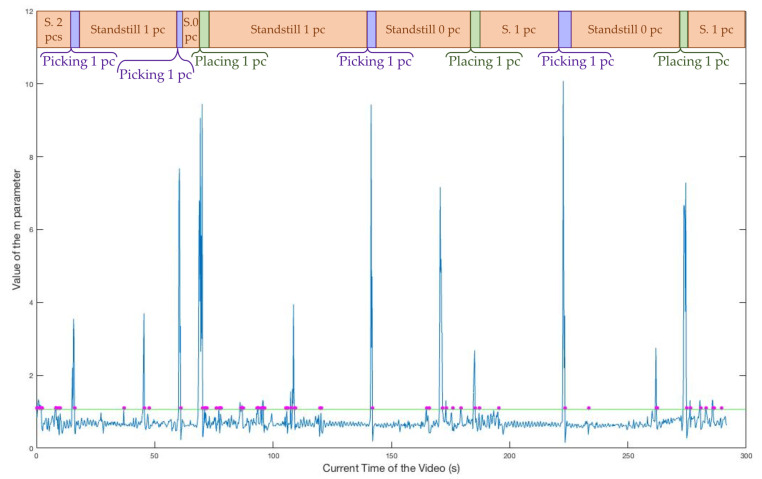
Plot of the m parameter regarding a video collected before the light ring installation.

The requirements (a), (b), and (c) are synthesized, from now on, with the name “Counting Capability”. Requirement (d) was named the “Real-Time Capability”, requirement, (e) “Responsiveness”, and (f) “Versatility”. As a general result, we also briefly resume the improvements resulting from the installation of the light ring, which is essential for application of the Motion Check procedure. In Figure 11, we show the behavior of the *m_t_* parameter for an old video, recorded before the light ring installation. The green horizontal line corresponds to the 1.08 threshold, and the magenta dots along this line correspond to every video frame which was further analysed.

The variance due to environmental shadows and natural lighting influences on the illumination of the framed table is evident, if we compare the m values with the timeline of events on the top of the figure. Indeed, the trend is not stable, even within stand-still phases, in contrast with the plot of the m parameter for a video recorded after the light ring installation (see Figure 4). The excessive fluctuation of *m_t_* results in an extremely higher number of frames further analysed (as indicated by the magenta dots) which, in turn, may affect the real-time and the correct-counting capabilities of the entire system. As a result of the light ring stabilizing the working conditions of the camera, there is no need to change the threshold once it is fixed and the behavior of *m_t_* is not influenced by shadows or natural light changes.

### 3.1. Testing Counting Capability

To test the “Counting Capability” of the two algorithms as objectively as possible, we decided to acquire videos in MP4 format and apply both algorithms offline to the same videos. In this way, we could be sure that possible imbalances of the performances achieved were only a matter of Counting Capability of the specific solution, and not caused by particular and strange behaviors of the operators under one real-time test that might differ from the behaviors analysed during the real-time test of the other algorithm. Specifically, given a video sequence, the processing algorithms could behave in four ways:

- correctly count one piece when the operator places an assembled one on the intermediate table (True Positive, TP);
- wrongly count when a piece has not been added on the table (False Positive, FP);
- correctly do not count when there have not been new pieces placed on the table (True Negative, TN); or
- wrongly do not count when an assembled piece has been placed on the table (False Negative-FN).

According to the definition of these four alternative outcomes and the performance evaluation criteria proposed in [33], we can define three metrics that summarize the capability of each of the two solutions in managing the counting task:

- Sensitivity, computed as the number of TP divided by the sum of the number of TP and FN, measures the solution’s capability of correctly identifying placed pieces and counting;
- Specificity, computed as the number of TN divided by the sum of TN and FP, measures the solution’s capability of correctly identifying the picked up pieces without counting; and
- Accuracy, computed as the sum of TP and TN divided by the sum of TP, FP, TN, and FN, measures the overall solution’s capability of correctly behaving.

In our case, we expect a comparable total frequency of occurrence of Negatives and Positives, given that we consider all the piece pick ups as Negative samples. Particularly, the recorded videos contained 88 piece pick ups and 90 piece placings, which were unconstrained and completely instinctual. We specify that they were unconstrained as, in our past work, we partially constrained the manner of picking up and placing objects to improve the previous algorithm’s performance.

To show the improvement resulting from the introduction of the Motion Check, we compared the two alternative solutions provided with the Motion Check procedure (proposed in this paper) to their original version (presented in our previous work). Thus, we prove the validity of the counting system with Motion Check, compared to that without. Additionally, by comparison with the improved versions, we prove the comparable performance of the simple and versatile Blob algorithm. In Table 1, we present the TP, TN, FP, and FN results regarding the offline test performed on videos collected after the installation of the light ring. Specifically, we present the results achieved by the two alternative algorithms, both in their original version where all of the frames were indiscriminately analysed (flowchart and details can be found in our groundwork [26]) and in their new versions provided with inter-frame Motion Check, as described in this work.

In Table 2, we report the values of the Sensitivity, Specificity, and Accuracy metrics for the two algorithms in both the original implementation and in the improved ones introduced in this work.

### 3.2. Testing Real-Time Capability

The aforementioned test could only provide information about the counting capability of the two alternatives once the video files were collected; however, the system should ensure its potential to analyse a continuous video stream without information loss over long times—at least for sixteen hours, given that the company is organized in two working shifts. To test this, we let the Real-time Counting App run on the Macbook for an entire day. As described before, the prototype App, while continuously processing frames, produces a log file containing timestamps for every count, frames further analysed, and even delays in frame capturing. If the interval between two consecutive frame grabbed is higher than 0.3 s, which means the algorithm is processing less than 3 frames per second, then the log file will list the timestamps of the *“Frame Capturing Delay”* error. By letting the application work for an entire work day and analysing the related log file, we could count the number of times in which the application was too slow in acquiring frames. We found only 1 occurrence of this kind of error in the log file within sixteen hours of continuous real-time processing using the blob-based algorithm for further processing, and 10 occurrences using the detector-based algorithm for further processing; proving that, being more computationally intensive, the latter alternative solution was implemented less successfully for real-time purposes.

### 3.3. Testing Other Requirements

As far as Versatility is concerned, we conducted an ad-hoc experiment using pieces which are different from the usual types assembled in the considered test assembly station. The only similarity between usual pieces and the ones used in this experiment was the chromatic characterization (they were partly black), while they differed in dimension and shape. The Improved Blob algorithm, with parameters regarding this new context of use which were easily and quickly defined using the Setting Tool App, demonstrated performances perfectly comparable with those reached during the Counting Capability Test with the usual product types presented in Section 3.1. On the other hand, the Improved ACF, which was trained on the original product type, made several mistakes, due to its unsuitability for the different objects placed on the table during this test. The adaptation of the detector to this new context is not as easy and fast as that for the adaptation of the blob-based solution, due to the mandatory necessity of training which, in turn, necessitates the development of a training image set. Therefore, the ACF proved to be unsuitable for plant-wide implementation.

**Figure 12:**
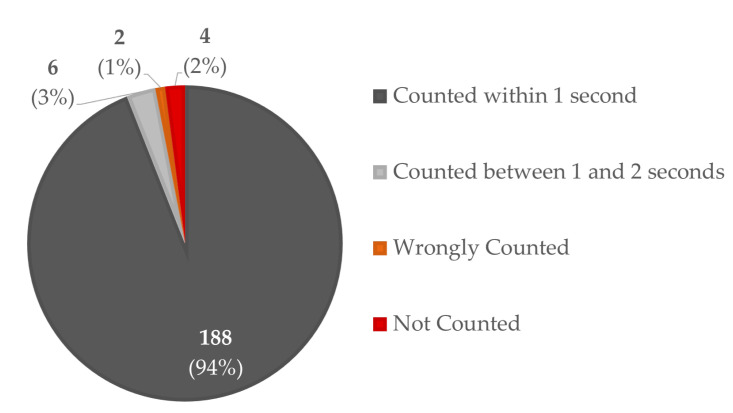
Results of the Real-time Counting Application Responsiveness test.

With regards to Responsiveness, we conducted additional live experiments using the Real-time Counting App, which allows the manual recording of the timestamps connected to the piece placing and autonomously saves the timestamps of the algorithm’s counting decisions. In this way, by simply comparing the two vectors of timestamps, we can compute the mean difference between the moment of placing and the moment of counting. In Figure 12, we summarized the results of this test done on 200 pieces.

It can be seen that 94% of pieces were correctly counted within 1 second after placing, which means almost simultaneously, while 3% of pieces suffered from a subtle delay of around 1 s. This may be due to the fact that the operator was very slow in taking their hand out of the framed area. The remaining 2% of pieces were not counted, these errors being due to the only eventuality that has not yet been addressed by our algorithms: when both operators simultaneously intervene in the ROI, one picking up and the other placing a piece, generating a balance if we analyze the number of objects present in the previously analysed frame and the number in the currently analysed frame.

## 4. Discussion

The final version of the solution is a complete system, which is able to process a video stream and count pieces placed as soon as they are left on the table. Relying on color-based processing and blob analysis, it is computationally fast and adaptable to every case in which a dark-coloured product is placed on a pale-coloured table. For the sake of completeness, we mention that it is easily adaptable to the opposite scenario, in which brighter products are placed on a dark table. Table 1 and Table 2 show a Sensitivity performance degradation concerning the Blob algorithm in its Original configuration, as compared to the Blob algorithm in the Improved configuration with preliminary Motion Check. This reduction is due to the only eventuality that the Motion Check cannot address, which is when the two operators simultaneously interfere in the framed area by placing and picking up at once. Indeed, the data collection was carried out without any sort of training for the assembly operators, who were left completely free to behave as they are used to. In order to implement this solution, the company must train their staff to avoid simultaneous interactions on the table. Original algorithms without Motion Check are error prone during the intervals in which one operator is either placing or picking up a piece. This reflects on the False Positive number of the Original Blob and, so, in both Specificity and Accuracy metrics. We can infer that, when using videos collected after the staff training, all the performance metrics of the Improved Blob should be equal or higher that the Original Blob’s metrics. Moving on to the best-performing solution, the ACF detector provided with the preliminary Motion Check procedure, we see that it had metrics only 1% higher than our proposed versatile and simple blob-based solution, which is why, for plant-wide implementation, we recommend the use of the blob-based algorithm. The solution is not yet absolutely reliable, as it commits some errors; however, its improvement from the first version is concretely noticeable, looking at Table 2. We aim to find other improvements for the processing algorithms, which allow it to reach very close to 100% in the Counting Capability metrics. We also wish to evaluate possible improvements for the Motion Check module and for the counting module of our solution, in both the detector-based and blob-based alternatives, and eventually find some other techniques which can be exploited for real-time counting.

## 5. Conclusions and Future Work

In this work, we proposed a Machine Vision algorithm which is able to analyze a video stream in real-time and automatically count the pieces assembled by an operator and placed on a table in a framed area. The developed algorithm integrates an inter-frame analysis mechanism which handles the human interactions in the framed area that can cause incorrect piece counting. In fact, after the development of a first solution in our preliminary paper, we identified the interaction of the operators with the framed area as a weakness of the previously developed algorithm. In order to overcome these limitations, we introduced the Motion Check phase as a preliminary step before conducting image processing, finalized at counting. This Motion Check is a novel adaptive examiner of motion which is not dependent on the specification of a fixed background, and understands whether there have been relevant movements between the current frame and the previous one. Using Motion Check and exploiting blob detection to identify the objects, the proposed solution was able to reliably count the pieces assembled by an operator. In fact, the proposed solution demonstrated very good performances, in terms of Sensitivity, Specificity, and Accuracy, when tested in a real situation in an Italian manufacturing firm’s shop floor. Moreover, the Real-time Capability, Responsiveness, and Versatility of our solution were evaluated in specific tests.

By analysing the frames corresponding to counting moments, we found that our improved algorithm counts when there are no hands in the framed area. Therefore, if we collect frames corresponding to counting moments, we automatically have a perfect and considerable data set for training a Machine Learning-based detector, or even a Deep Learning based one. We envision the possibility of merging the basic blob-based solution as a preliminary automatic way for developing a more robust detector-based solution for every station and for every assembly line. With this insight, we aim to simplify the development of an advanced Machine vision detector for custom object recognition purposes, even for non-experts. Another point we want to address is that, with the presented configuration of the solution, we analyse between 3 and 10 fps; nonetheless, we would like to improve the frame rate, speeding up computation by implementing the algorithms directly on a dedicated hardware platform provided with FPGA and GPU. This goes together with a need for system optimization, in order to ensure the correct and real-time analysis of multiple converging video streams. Coherently, source coding and communication protocols have to be optimized and tested for the design of a system architecture which exploits either edge computing or cloud computing for carrying out the simultaneous and continuous processing of several and parallel video streams, which is the final objective of the company.

## Figures and Tables

**Table 1:** Summarized results for the blob-based and the ACF-based original algorithms, as well as for the improved blob-based and improved ACF-based algorithms.

	Original Blob		Original ACF		Improved Blob		Improved ACF	
	Positive	Negative	Positive	Negative	Positive	Negative	Positive	Negative
True	90	75	80	85	88	86	87	85
False	13	0	3	10	2	2	3	3

**Table 2:** The three performance metrics computed on the results reached by the two algorithms, both in their old version and in the new one with Motion Check and somemodifications.

	Original Blob	Original ACF	Improved Blob	Improved ACF
Sensitivity	100%	89%	96.7%	97.8%
Specificity	85.2%	96.6%	96.6%	97.7%
Accuracy	92.7%	92.7%	96.6%	97.8%

## Abbreviations

The following abbreviations are used in this manuscript:

CV	Computer Vision
MV	Machine Vision
ROI	Region Of Interest
ACF	Aggregated Channel Features
GUI	Graphical User Interface
